# Tumor-suppressive effects of atelocollagen-conjugated hsa-miR-520d-5p on un-differentiated cancer cells in a mouse xenograft model

**DOI:** 10.1186/s12885-016-2467-y

**Published:** 2016-07-07

**Authors:** Yoshitaka Ishihara, Satoshi Tsuno, Satoshi Kuwamoto, Taro Yamashita, Yusuke Endo, Keigo Miura, Yugo Miura, Takemasa Sato, Junichi Hasegawa, Norimasa Miura

**Affiliations:** Division of Pharmacotherapeutics, Department of Pathophysiological & Therapeutic Science, Faculty of Medicine, Tottori University, 86 Nishicho, Yonago, Tottori 683-8503 Japan; Division of Molecular Pathology, Faculty of Medicine, Tottori University, 86 Nishicho, Yonago, Tottori 683-8503 Japan; Department of Gastroenterology, Tottori University Hospital, 86 Nishicho, Yonago, Tottori 683-8504 Japan; PEZY-Pharma, 86 Nishicho, Yonago, Tottori 683-8503 Japan; Orthopedic Surgery, Tokyo Medical and Dental University, 1-5-45 Yushima, Bunkyo-ku, Tokyo 113-8510 Japan; Division of Neurobiology, School of Life Science, Faculty of Medicine, Tottori University, 86 Nishicho, Yonago, Tottori 683-8503 Japan

**Keywords:** Cancer, Atelocollagen, miR-520d-5p, Xenograft model, Therapeutic effect

## Abstract

**Background:**

We previously demonstrated that hsa-miR-520d-5p can convert cancer cells into induced pluripotent stem cells (iPSCs) or mesenchymal stem cells (MSCs) via a demethylation process and p53 upregulation in vivo. Additionally, we have reported the non-tumorigenic effect of miR-520d-5p on normal human cells, including fibroblasts.

**Methods:**

We used atelocollagen-conjugated miR-520d-5p (520d/atelocollagen) to confirm the possibility of a therapeutic effect on cancer cells. We traced the size and signal intensity of GFP-expressing tumors in mice each week, beginning 4 weeks after subcutaneous inoculation.

**Results:**

520d/atelocollagen treatment suppressed tumor growth by greater than 80 % each week relative to controls and resulted in an approximately 30 % disappearance of tumors. In mice whose tumors disappeared, the existence of human genomic material at the injection site was examined by quantitative Alu-PCR, and we confirmed the co-existence of both species-derived cells. In every site where a tumor disappeared in immunodeficient mice, GFP protein was expressed in the connective tissues, and approximately 0.1 % of the extracted DNA contained human genomic material. We could not identify any adverse effects in vivo.

**Conclusions:**

This is the first report to confirm an inhibitory effect of 520d/atelocollagen on cancer cells in vivo. The development of optimized modifications of this carrier is expected to enhance the efficiency of entry into tumor cells and the induction of its inhibitory effect.

**Electronic supplementary material:**

The online version of this article (doi:10.1186/s12885-016-2467-y) contains supplementary material, which is available to authorized users.

## Background

Mature miRNAs are endogenous, small, noncoding RNA (ncRNA) molecules that are 18 to 23 nucleotides in length and transcribed by RNA polymerase II. Mature miRNAs function as post-transcriptional gene regulators, controlling gene expression in many cellular processes [1, 2] or mediating cellular differentiation, reprogramming and the initiation and progression of human cancer [3–5]. Alterations in miRNA expression levels influence tumor growth by modulating the functional expression of target genes that are implicated in the regulation of tumor cell apoptosis or proliferation [6]. The central role of miRNAs during tumorigenesis has been investigated in different tumor types, including melanomas, hepatomas, brain tumors, and breast tumors [7–9].

We previously identified an RNA gene (*RGM249*) that may indirectly regulate human telomerase reverse transcriptase (hTERT) expression in undifferentiated cancer cell lines via the induction of double-stranded (ds), small interfering (si), and short hairpin (sh) RNAs. *RGM249* is implicated in the regulation of cell development, cell differentiation, anti-inflammatory effects, and cell growth in undifferentiated cancers [10]. Our previous report demonstrated that siRNAs against three small RNAs derived from ncRNA in vivo affected the metastatic or proliferative abilities of cells during in vivo tumor growth [11].

RNA-based therapeutics for cancer have been developed. For example, viral vectors, non-viral reagents, and nanoparticles have been pre-clinically and clinically tested to overcome their shortcomings, such as instability in vivo and inefficient and inaccurate targeting of organs or tumor tissues [12–18].

We have also reported on the safety, efficacy, and specificity of drug delivery systems (DDSs) using gelatin hydrogel microspheres or atelocollagen for subcutaneous (s.c.) injection and spermine-pullulan or atelocollagen for intravenous administration [19–23]. We therefore aimed to elucidate the physiological functions of miRNA-like molecules generated from *RGM249* and their roles in carcinogenesis, differentiation, and pluripotency. We also investigated their potential utility for antitumor therapy or regenerative medicine in vivo.

Targeting by miRNAs is accomplished via base-pair interactions between the 5′ end of miRNAs and sites within the coding and/or untranslated regions (UTRs) of gene transcripts; target sites in the 3′ UTR lead to more effective translational dysfunction [24, 25]. Because a specific miRNA generally targets at least hundreds of different mRNAs, it is extremely difficult to elucidate miRNA regulatory pathways [26]. In the case of miR-520d-5p, which can convert undifferentiated hepatoma cells (HLF) to a benign or normal status in vivo [27], available bioinformatics predict that it has greater than 8000 target genes. Because miR-520d-5p did not appear to have any toxic effects on normal cells or cancer cells and did not induce tumorigenicity or malignant transformations in vivo [28], we examined the therapeutic effects of 520d-5p conjugated with atelocollagen as a drug delivery system (DDS) carrier on undifferentiated cancer cells using in vivo imaging.

## Methods

### Atelocollagen

Atelocollagen is a highly purified type I collagen of the calf dermis treated with pepsin (Koken Co., Ld, Tokyo, Japan).

### RNA preparation

**Fig. 1:**
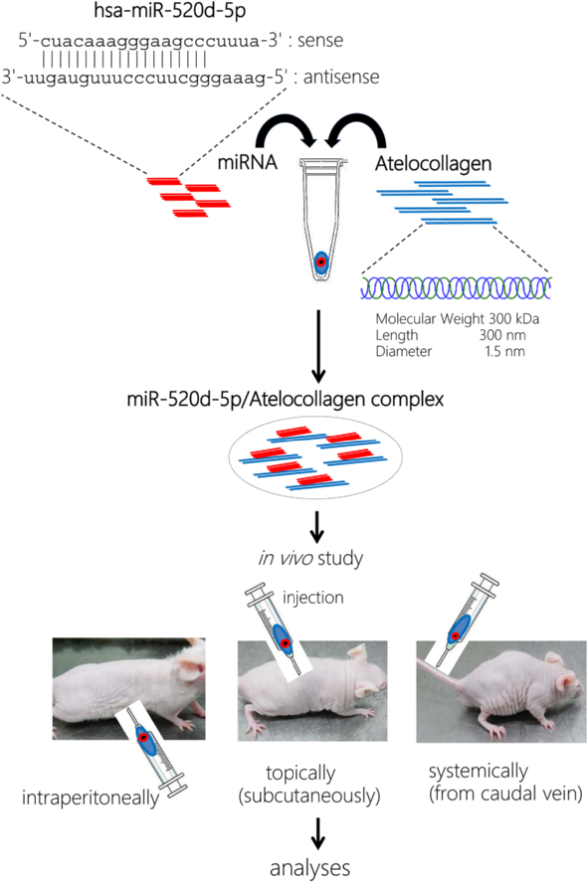
Schematic of the study design and processes. Atelocollagen and miR-520d-5p were conjugated following the manufacturer’s instructions, and the resulting complex was injected into immunodeficient mice that were inoculated with cancer cells. In addition, 520d-conjugated atelocollagen was administered to mice via three different methods (see Methods for further details)

Synthetic 20-nt RNAs were purchased from Koken (Tokyo, Japan) in deprotected, desalted and annealed form. The sequence of our prepared hsa-miR-520d-5p was 5′-cuacaaagggaagcccuuuc-3′ and 3′-uugauguuucccuucgggaaag-5′ (Fig. 1). A non-specific control miRNA duplex was purchased from HSS (Sapporo, Japan), and the scrambled sequence was 5′-gaguccgccucuauagacaa-3′.

### Formation of the miRNA/atelocollagen complex

The miRNAs and atelocollagen complexes were prepared according to the manufacturer’s instructions (Koken Co.). Briefly, equal volumes of 1.0 % atelocollagen [in phosphate-buffered saline (PBS) at pH 7.4] and miRNA solution (in PBS) were combined and mixed by rotation (4 rpm) at 4 °C for 20 min before centrifugation (10,000 rpm) at 4 °C for 1 min. The complex was used for inoculation into immunodeficient mice (KSN/Slc) (Shimizu Laboratory Supplies Co., Ltd., Kyoto, Japan). The final concentrations of atelocollagen for subcutaneous injection, intraperitoneal injection or intravenous injection in vivo were 10 μM, 40 μM or 0.5 %, respectively (Fig. 1).

### Stability of the siRNA/atelocollagen complex

Aliquots of 0.9 mg of miRNA and 0.5 % atelocollagen complexes were incubated in the presence of 0.1 mg/ml RNase A (NipponGene, Tokyo, Japan) for 0, 5, 15, 30, 45 and 60 min at 37 °C. The solutions were then extracted with phenol and phenol/chloroform/isoamyl alcohol (25:24:1). The miRNAs were precipitated with ethanol, separated by 3.5 % agarose gel electrophoresis and visualized by ethidium bromide staining (Fig. 1).

### Cell lines and immunodeficient mice

Two undifferentiated human cell lines, hepatoma cells (HLF) and malignant melanoma cells that do not produce melanin (HMV-1) were obtained from Tohoku University and the American Type Culture Collection (ATCC), respectively. They were maintained in Roswell Park Memorial Institute (RPMI) 1640 Medium (WAKO, Tokyo, Japan) with 10 % heat-inactivated fetal bovine serum (FBS) at 37 °C in a humidified atmosphere of 5 % CO_2_. A total of 5 × 10^7^ cells were harvested and inoculated into mice subcutaneously, intraperitoneally or intravenously using a 26-gauge needle. Six-week-old immunodeficient mice were inoculated for in vivo study by miR-520d-5p transfection. Immunodeficient KSN/Slc mice were used to examine the anti-metastatic effects of miR-520d-5p or the transformation to normal status by miR-520d-5p (Fig. 1). The athymic mice were anesthetized by intraperitoneal injection of 100 mg/kg Nembutal. All animals were housed and fed in the Division of Laboratory Animal Science at Tottori University (Tottori, Japan) under a protocol that was approved by the Japanese Association for Accreditation for Laboratory Animal Care, and animal research and handling were performed in strict accordance with the federal Institutional Animal Care and Use Committee guidelines. All experiments reported in this study were approved by an institutional committee (#13-Y-37, #18-2-41, #h27-074) and ARRIVE guidelines have been followed.

In addition, the human mesangial cell line 293FT (Invitrogen Japan K.K., Tokyo, Japan) was used for producing GFP-expressing lentiviral particles. Briefly, 293FT cells were cultured in DMEM supplemented with 10 % FBS, 0.1 mM MEM non-essential amino acids solution, 2 mM L-glutamine and 1 % penicillin/streptomycin.

### Gene expression analysis by reverse transcription-polymerase chain reaction (RT-PCR)

Total RNA, including the small RNA fraction, was extracted from cultured cells or from homogenized mouse tissues with the mirVana miRNA Isolation Kit (Ambion, Austin, TX, USA). Mature miRNA (miR-520d-5p, 25 ng/μl) was quantified with the Mir-X™ miRNA qRT-PCR SYBR® kit (Takara Bio, Tokyo, Japan) according to the manufacturer’s instructions. The gels were run under the same experimental conditions. PCR and data collection analyses were performed with a BioFlux LineGene (Toyobo, Nagoya, Japan). To analyze the RT-PCR results, all data were normalized to a β-actin internal control. U6 small nuclear RNA was also used as an internal control. Total RNA (25 ng/μl) was reverse transcribed and amplified using the KAPA SYBR FAST One-Step qRT-PCR Kit (Nippon Genetics Co, Ltd, Tokyo, Japan). RNA quantification was confirmed by sequencing with high reproducibility. Additional file 1: Table S1 presents the primer sequences that were used for mRNA or miRNA quantification. The data were analyzed statistically by one-way analysis of variance (ANOVA) or Mann-Whitney *U* tests, and significant differences are shown as *P < 0.05 and **P < 0.01.

### Cell migration assay

The invasive abilities of transfected cells were estimated using a CIM-Plate 16, which detects cell invasion/migration in real time following the manufacturer’s instructions (xCELLigence system, Roche, Basel, Switzerland).

### Immunocytochemistry

Immunohistochemical examination was performed with antibodies to detect engraftment evidence (anti-hSIX1 and anti-hGFAP) following the manufacturer’s instructions (Atlas Antibodies AB, Stockholm, Sweden and R&D Systems, Minneapolis, MN, USA, respectively). HLF and HMV-I cells were infected with lentiviral particles that contained hsa-miR-520d-5p. Floating transfectants were harvested and transferred to new culture dishes for microscopic examination or slide chambers for immunostaining.

### Flow cytometry analysis

Cell cycle analyses were conducted to confirm that the 520d/HMV-I cells comprised benign cell populations, as previously reported for 520d/HLF [27]. DNA content of the cells was analyzed with a flow cytometer (Epics Altra; Beckman Coulter Inc., CA, USA). Cells were assessed by approximately 20,000 collected events after the transfection of a pMIRNA1-miR-520d-5p/GFP clone using EXPO32 ADC analysis software. GFP-positive cells were sorted on a Moflo XDP cell sorter (Epics Altra; Beckman Coulter Inc.). Specifically, for cell cycle analysis, a single cell suspension was washed once with cold PBS. The cell pellet was loosened by shaking the tube gently and was fixed with 3.7 % formalin in ddH_2_O, which was added dropwise. The cells were then incubated at least overnight at 22 °C. After fixation, the cells were washed twice with cold PBS to remove the EtOH, resuspended at 1 × 10^6^ cells/ml in PBS with 100 U/ml RNase A and incubated for 50 min at 37 °C. Next, 50 mg/ml of propidium iodide was added, and the mixture was incubated for 40 min on ice in the dark. DNA content was analyzed with a flow cytometer; the cells were assessed by approximately 20,000 collected events after the transfection of a pMIRNA1-miR-520d-5p/GFP clone and prior to EXPO32 ADC analysis software.

### miRNA transfer procedures in vivo

The miR-520d-5p/atelocollagen complex was injected into immunodeficient mice topically, intraperitoneally or systemically (Fig. 1). To identify the anti-cancer effects of miR-520d, we examined its effects on two cell lines (HLF or HMV-I) in vivo. Atelocollagen was used as a carrier. The cells were subcutaneously inoculated into the right flanks of the mice (*n* = 8 for each cell line), intraperitoneally into the center of the abdomen (*n* = 8) or intravenously into the caudal vein (*n* = 8). Because miRNA expression in tumors was evaluated after categorizing the parts of the tumor as described in our previous report [11], we started examining the therapeutic effect when the tumor size was 8 mm (for HLF) or 5 mm (for HMV-I) in the subcutaneous xenograft model. The miRNA/atelocollagen conjugates were administered to 6-week-old mice every 7 days for approximately 90 days. The observation period was a maximum of 6 months for mice whose inoculated tumors disappeared. The animals were sacrificed after 15 weeks (for HLF) or 12 weeks (for HMV-I) and examined for gross tumor formation and metastasis. Mice prepared for systemic delivery received an intravenous injection every week, starting 1 week after the first injection of HLF or HMV-I cells. The injection volume was 200 μl and contained 1 × 10^7^ cells. Palpable tumors were confirmed on day 7 following inoculation. Volume estimations were determined using the following formula: volume = π/6 × width × length × height. After the mice were sacrificed, we assessed intrahepatic tumors, intraperitoneal dissemination, micrometastases in organs (brain, lung, heart, spleen, small intestine, liver and kidney) and the presence or absence of atelocollagen-associated adverse effects, such as intravascular embolism. In the intraperitoneal xenograft model, therapeutic injection was initiated 1 week after the inoculation at the same concentration of 520d/atelocollagen as of the subcutaneous injection. Via systemic administration through the caudal vein, 0.5 % 520d/atelocollagen was used 1 week later or earlier than the inoculation. Intratumoral gene expressions were estimated according to our previously reported method [11].

### Evaluation of miRNA transfer in vivo

RNA quantification was confirmed by sequencing with high reproducibility. Tissues were subjected to miRNA extraction using a mirVana miRNA Isolation kit, and miRNA expression was examined using a Mir-X™ miRNA qRT-PCR SYBR kit to confirm suppression by siRNA and to evaluate changes in miRNA expression using the 2_-ΔΔCt_ method according to the manufacturer’s recommendations. Genes were chosen from those with altered expression levels in response to miR-520d-5p induction and silencing. Western blot analyses were performed using the i-Blot gel transfer system (Life Technologies Japan, Tokyo, Japan). Antibodies [anti-p53, anti-Nanog, anti-Oct4, and anti- activation-induced cytidine deaminase (AICDA)] were diluted 1:500, and the control anti-β-actin antibody was diluted 1:1000. Chemiluminescent signals were detected within 1 min using a LAS-4000 (Fujifilm, Tokyo, Japan).

### Analysis of miRNA delivery using in vivo imaging

Cells were subcutaneously injected (5 × 10^7^ cells per site) into athymic nude mice. When the tumors grew to 5 to 8 mm in size, atelocollagen alone, control miRNA conjugated with atelocollagen (scrambled), or 520d-5p/atelocollagen was injected around the tumors as described previously [11]. Each group consisted of 8 animals. In vivo bioimaging was conducted on a cryogenically cooled IVIS system (Xenogen Corp., Waltham, MA, USA) using LivingImage acquisition and analysis software [29]. Tumor growth was monitored by measuring the light emission from individual mice 21 to 28 days after miRNA administration. Tumor growth was not affected by these treatments.

### Histological examination

Tumor nodules were investigated macroscopically or under a dissecting microscope with bright-field imaging. The tissue samples were fixed in 10 % buffered formalin overnight, washed with PBS, transferred to 70 % ethanol, embedded in paraffin, sectioned and stained with hematoxylin-eosin (HE). GFP expression in tissues was observed in unstained and HE-stained samples using an EVOS FL cell imaging system (Life Technologies Japan, Tokyo, Japan).

### Lentiviral vector construct and reporter gene labeling of tumor cells

To examine the effects of miR-520d-5p overexpression, we transfected pMIRNA1-miR-520d-5p/GFP (20 mg; System Biosciences, Mountain View, CA, USA) into 293FT cells (5 × 10^6^ cells/10-cm culture dish). To harvest viral particles, the cells were centrifuged at 170,000 x *g* (120 min, 4 °C). The viral pellets were collected, and viral copy numbers were measured with a Lenti-X™ qRT-PCR Titration kit (Clontech, Mountain View, CA, USA). For infection into HLF cells or HMV-I cells, 1 × 10^6^ lentiviral copies were used per 10-cm culture dish. To estimate the efficacy of infection by the GFP-expressing lentiviral vector (pMIRNA1/GFP, 20 μg; System Biosciences, Mountain View, CA, USA), GFP expression in HLF or HMV-I cells was detected with the EVOS FL cell imaging system. Transfectants without viral particles in the culture supernatant were used for tumor formation in vivo.

### Quantitative Alu-PCR

Extraction of DNA from tumor tissues or injected sites without tumors was performed using CellEase Tissue II according to the manufacturer’s instructions (Biocosm Inc., Tokyo, Japan). By quantitative Alu-PCR [30, 31], the presence of human-derived genomic DNA in 50 ng of DNA extracted from the injection site of mice was examined (*n* = 4). The subcutaneous tumor disappeared in all cases, and the scar-like, slightly protruding parts were examined. Primer sequences were as follows: sense, 5′-GTCAGGAGATCGAGACCATCCC-3′; and antisense, 5′-TCCTGCCTCAGCCTCCCAAG-3′. Thermal cycling was conducted at 95 °C for 2 min (“hot start”), followed by 35 cycles of 95 °C for 15 s, 68 °C for 30 s and 72 °C for 30 s. Initial experiments varied the annealing and/or extension times and temperatures to optimize the assay. A melt curve was also performed after the assay to verify the specificity of the reaction. The melt curve consisted of 20 s at 72 °C followed by a ramp up of 1 °C steps with a 5-s hold at each step.

### Immunohistochemical staining

Specimens were fixed in 4 % paraformaldehyde for 7 days. Paraffin sectioning was conducted using a Young-type sliding microtome (Sakura Finetek Japan, Co., Ltd.) with a disposable microtome blade. Sections were approximately 3 μm in thickness and were mounted on silane-coated slides. Immunohistochemical staining was performed according to the labeled streptavidin-biotin (LSAB) method. After the deparaffinized sections had been autoclaved at 120 °C for 10 min to block endogenous peroxidase activity, they were incubated sequentially at 4 °C for 1 h each with anti-ZO-1 (Zymed) diluted to 1:100 in Tris-buffered saline (TBS) and then in streptavidin-biotin-peroxidase solution according to the instructions for the LSAB kit (Dako Japan Co., Ltd.). The immunoreaction was visualized with the peroxidase-diaminobenzidine (DAB) reaction. Finally, the sections were counterstained with hematoxylin. The stained sections were then observed with an Olympus DP70 (Olympus, Japan) light microscope. The antibodies (SIX1 and GFAP) were diluted at 1:500 for use.

### Statistical analyses

The Mann-Whitney *U*-test was used for comparisons between the controls, the scrambled and/or the miR-520d-5p results with one observed variable. *P*-values considered to indicate a significant difference were labeled as follows: *, *P* < 0.05 and **, *P* < 0.01. In box plots, the top and bottom of each box represent the 25th and 75th percentiles, respectively, thus providing the interquartile range. The line through the box indicates the median, and the error bars indicate the 5th and 95th percentiles.

## Results

### In vitro study in undifferentiated melanoma cells (HMV-I)

**Fig. 2:**
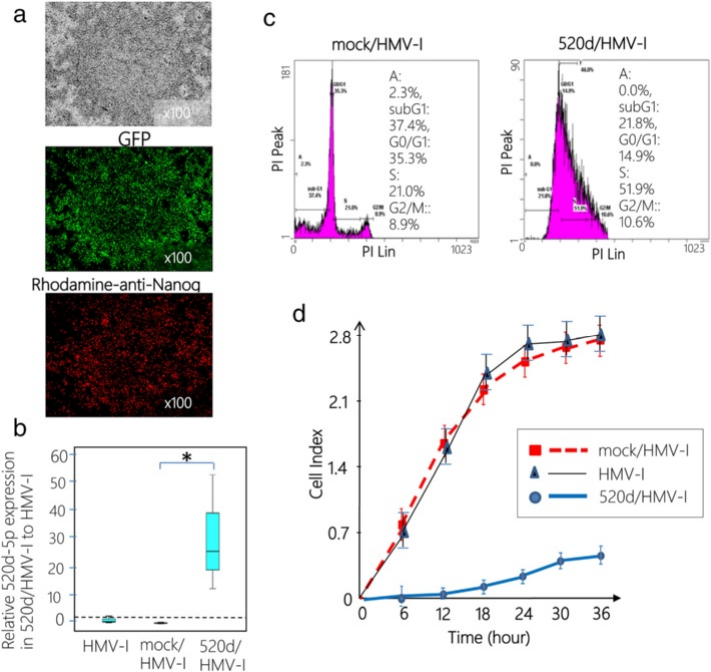
Lenti-viral transfection of 520d-5p to HMV-I cells in vitro. **a** A representative phenotype of 520d/HMV-I is presented (x100 magnification). Spheroid-like cells comprising a small fraction of the cell population are presented (*top*). Analysis of GFP expression confirmed the effective induction (more than 99 %) of 520d/HMV-I cells (x100 magnification) (*middle*). miR-520d-5p induced the expression of Nanog in HMV-I cells (x100 magnification) (*bottom*). The phenotypic changes were not sufficient to assess whether they were malignant or benign. **b** Significant 520d-5p expression was confirmed in 520d/HMV-I cells (*, *P* < 0.01 by the Mann-Whitney *U* test). The relative expression of 520d-5p in 520d/HMV-I cells compared with mock/HMV-I cells is presented. All expression data are standardized to the β-actin expression level (*n* = 4). **c** Fluorescence activated cell sorting (FACS) analysis revealed that GFP-positive 520d/HMV-I cells (*right*) exhibited increased DNA content in the S phase compared with GFP-positive mock/HMV-I cells (*left*). **d** The invasive abilities of HMV-I, mock-transfected HMV-I (mock/HMV-I) and HMV-I cells treated with miR-520d-5p (520d/HMV-I) were estimated with a migration assay using a fibronectin membrane (10 μg/ml). Most of the 520d/HMV-I cells did not pass through the membrane

As we reported previously [27], HLF could be converted to a benign or normal status by miR-520d-5p via a stemness-mediated process. In the present study, we assessed the tumor-suppressive effect of miR-520d-5p on undifferentiated melanoma cells (HMV-I) in vitro and attempted to examine the therapeutic effect of the miRNA on two cell lines (HLF or HMV-I) using atelocollagen as a DDS carrier biomaterial. Although miR-520d-5p completely penetrates all of the tumor cells in tumor tissues, viable cells persist after its delivery. We transfected HMV-I cells with 520d-5p using a lentiviral vector in vitro (520d/HMV-I) and confirmed the formation of spheroid-like populations (Fig. 2a; top) that expressed the GFP reporter gene (Fig. 2a; middle) and the pluripotent marker Nanog (Fig. 2a; bottom). After we confirmed miR-520d expression (Fig. 2b), DNA content, analyzed by flow cytometry, revealed a shift toward homogeneous proliferation in the S phase, as described for the HLF cells in our previous report [27] (Fig. 2c). The 520d/HMV-I cells did not invade the fibronectin membrane in a migration assay, as was observed for the mock/HMV-I or HMV-I cells (Fig. 2d). A similar experiment was performed in HLF cells [27].

### In vivo study in two undifferentiated cells (HMV-I and HLF)

**Fig. 3:**
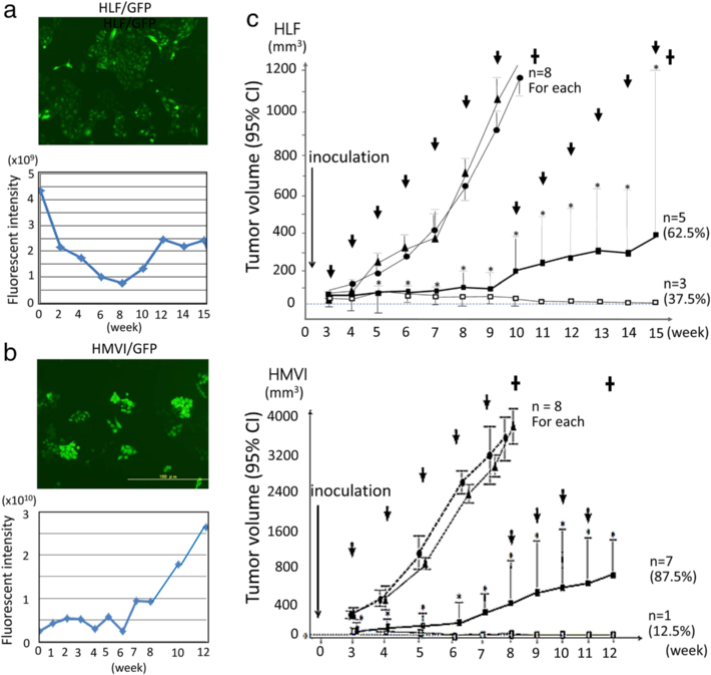
An in vivo study to assess the topical effects of 520d-5p-conjugated atelocollagen on cancer tissues. **a**, **b** After we generated GFP-expressing HLF (HLF/GFP) or HMV-I (HMV-I/GFP) cells using lentiviral constructs (*top for each*), the therapeutic effects of 520d/atelocollagen on the resulting GFP-expressing tumors in mice were examined. The average fluorescence intensity in tumors was measured, and representative data are presented through week 8 (*bottom of each panel*). GFP expression was maintained during the observation period in both studies. *Dotted lines* indicate tumor growth in controls. **c** Overviews of the in vivo study using 520d/atelocollagen in a mouse xenograft model (*top*, HLF; *bottom*, HMV-I). In the group that was administered the 520d/atelocollagen complex, tumor volume was significantly suppressed relative to the control group. *, *P* < 0.01 by the Mann-Whitney *U* test. The tumor volume of HLF cells from 11 to 15 weeks or of HMV-I cells from 9 to 12 weeks was analyzed and compared with control or scrambled data at 10 weeks or 8 weeks, respectively. The *arrow* indicates the timing of the doses that were administered to each group from 3 to 9 weeks (c) and from 3 to 7 weeks (d). **↓**: injection of complex (once a week); ▲: atelocollagen alone; ●: scramble/atelocollagen; ■: miR-520d-5p/atelocollagen (cases with suppressive growth); □: miR-520d-5p/atelocollagen (cases with tumor disappearance); ✝: mice were sacrificed; CI: confidence interval

We performed an in vivo study using 520d-5p-conjugated atelocollagen (520d/atelocollagen) to assess its topical effects on cancer tissues after we confirmed intense GFP expression in the two cell lines (Fig. 3a top, b top). Although GFP expression was confirmed, the intensity of expression was somewhat variable between cells (Fig. 3a, b left bottom, respectively). After subcutaneously inoculating HLF (top) or HMV-I (bottom) into nude mice and allowing the tumor to develop to 5 to 8 mm in size (corresponding to the time of approximately 3–4 weeks after inoculation), 520d/atelocollagen was injected into the tumor once per week. The average fluorescence intensity after subcutaneous injection of 520d/atelocollagen was recorded and is depicted in Fig. 3a and b (bottom). This analysis revealed that 62.5 % (5/8) of HLF tumors and 87.5 % (7/8) of HMV-I tumors were significantly suppressed, whereas 37.5 % (3/8) of HLF tumors (Fig. 3c; top) and 12.5 % (1/8) of HMV-I cells (Fig. 3c; bottom) disappeared. The tumors that eventually disappeared were observed for 12 weeks. During that time and in the absence of 520d/atelocollagen injections, we did not detect any recurrent tumors (Additional file 2: Figure S5).

Among HLF tumors, the average suppression rates of tumor size in the subcutaneous, intraperitoneal, and systemic (metastasis) models were 92.7 %, 75.0 % (6/8) and 100 % (8/8), respectively (Table 1). An average of 85.9 %, 87.5 % (7/8) or 100 % (8/8) of HMV-I tumors exhibited tumor size suppression in the subcutaneous, intraperitoneal, and metastasis models, respectively (Table 1). The suppressive effect of miR-520d-5p on subcutaneous tumors and metastasis was calculated based on the data collected each week or the findings at the time of sacrifice (Additional file 3: Table S2 or Additional file 4: Table S3, respectively). However, when the animals were sacrificed, most tumors grew increasingly, and the suppressive effect of 520d-5p on tumor growth could not be observed by RT-PCR (Additional file 5: Figure S6).

**Table 1:** Summary of therapeutic effect miR-520d-5p/Atelocollagen complex in a xenografted model

Cancer cells	Tumor suppressive effect in subcutaneous model (*n* = 8 for each) 10 week, average%	Therapeutic effect in peritoneal model (*n* = 8 for each) % (effective/total)	Metastasis suppressive effect in caudal vein model (*n* = 8 for each) % (effective/total)
HLF (undifferentiated hepatoma)	92.7	75.0 (6/8)	100 (8/8)
HMV-1 (undifferentiated melanoma)	85.9	87.5 (7/8)	100 (8/8)

effective/total: the number of mice with tumor disapperance/total number of examined mice

10 week, average%: average of tumor suppressive rate each week compared with control during 10 week obsevation (*see* Additional file 7: Figure S2)

### In vivo imaging using GFP expression in tumors

**Fig. 4:**
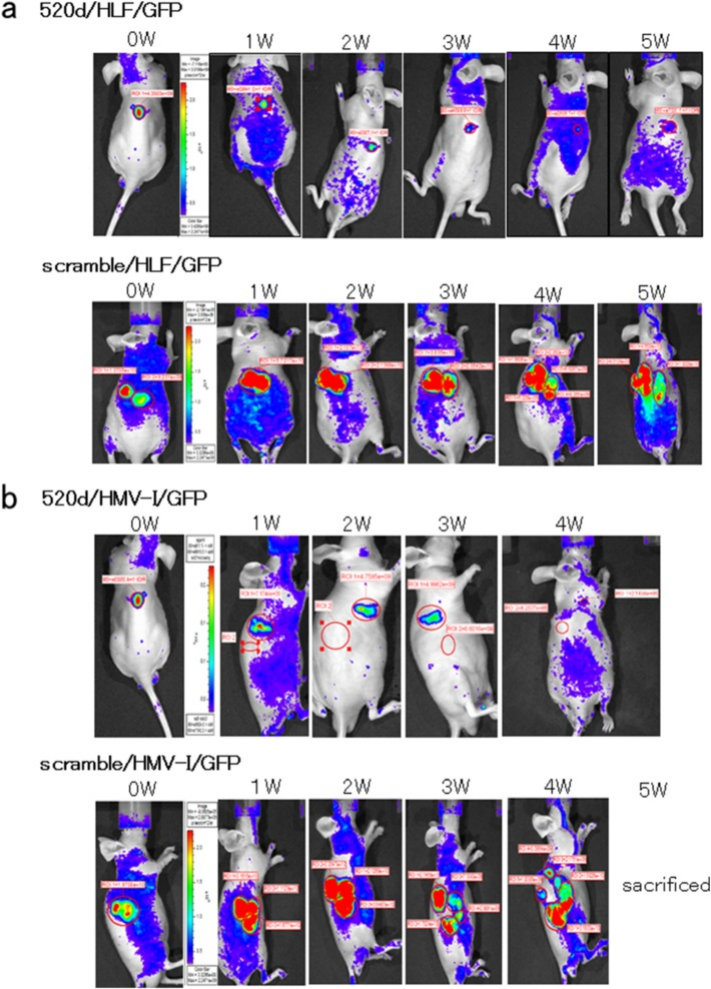
The therapeutic effect of 520d-5p-conjugated atelocollagen on subcutaneously inoculated tumors as monitored by an in vivo imaging system. **a** A representative image of the therapeutic effect of 520d-5p/atelocollagen using an in vivo imaging system. At each inoculation site, 37.5 % (3/8) of the tumors disappeared. The growth of tumors that received 520d-5p was significantly suppressed, and metastatic ability was entirely inhibited in the remaining mice. A time course (*top*) of 520d/HLF/GFP cells (520d-5p-transfected and GFP-expressing HLF) is depicted. Representative sequential images (*bottom*) of scrambled/HLF/GFP cells (scrambled-transfected GFP-expressing HLF). **b** In HMV-I tumors, 12.5 % (1/8) of the tumors disappeared. After treatment with 520d-5p, the growth of other tumors was significantly suppressed, and their metastatic ability was entirely inhibited. 520d/HMV-I/GFP and scrambled/HMV-I/GFP indicate 520d-5p-transfected, GFP-expressing HLF (*top*) and scramble-transfected, GFP-expressing HMV-I (*bottom*), respectively

The therapeutic effect of 520d-5p on subcutaneously inoculated tumors was evaluated based on the intensity of the GFP signal using *an* in vivo imaging system. Among the GFP-expressing HLF cells that received 520d-5p (520d/HLF/GFP), 37.5 % (3/8) exhibited a gradual decrease in the signal, followed by disappearance of the signal in the 5th week (representative data are shown in Fig. 4a top), whereas GFP-expressing HLF cells that received a scrambled construct (scrambled/HLF/GFP) exhibited an increase in the GFP signal accompanied by gradual growth (representative data are shown in Fig. 4a bottom). Among GFP-expressing HMV-I cells that received 520d-5p (520d/HMV-I/GFP), 12.5 % (1/8) exhibited a gradual decrease in the GFP signal followed by its disappearance in the 5th week (representative data are shown in Fig. 4b top), whereas GFP-expressing HMV-I cells that received the scrambled construct (scrambled/HMV-I/GFP) exhibited an increase in GFP signal accompanied by gradual growth (representative data are shown in Fig. 4b bottom).

### Evaluation of tumor suppression by histological and molecular examination

**Fig. 5:**
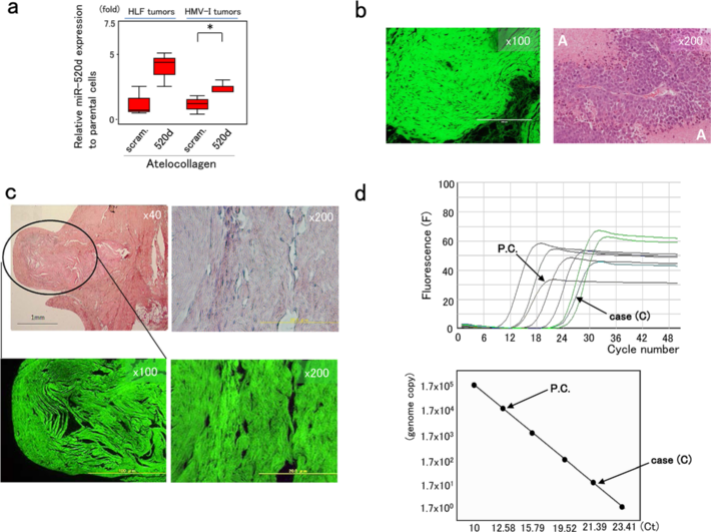
Evidence of human genomic DNA at the injection site. **a** miR-520d-5p expression was examined in HLF (*n* = 5) and HMV-I (*n* = 7) tumors resected after 520d/atelocollagen treatment. 520d/HMV-I tumors significantly expressed 520d-5p (*, *P* < 0.05), and 520d/HLF tumors tended to express miR-520d-5p (*P* = 0.051). scram.: scrambled. **b** Microscopic examination of HLF (*left*; HE stain, x100 magnification) and HMV-I tumors (*right*; HE stain, x200 magnification) that displayed suppressed tumor growth. HLF tumors treated with 520d/atelocollagen exhibited GFP-expressing muscle tissues, including undifferentiated tumor cells unaccompanied by necrosis. HMV-I tumors treated with 520d/atelocollagen showed undifferentiated neoplastic cells. This finding is not inconsistent, as malignant melanomas are observed to be accompanied by extensive necrosis, as shown around A. **c** Histological findings in the area where there should have been a tumor are presented (HE stain, x40 magnification). The area of the HLF tumors that disappeared is surrounded by an oval (*left top*). GFP expression was confirmed (*left bottom*). Histological findings in the disappearance site of HMV-I tumors showed no malignant cells. This finding was similar to that for 520d-treated HLF tumors (HE stain, x200 magnification) (*right top*), but we observed GFP even in the connective tissue (*right bottom*). We did not observe any malignant cells in the injection sites of mice in either case. **d** Fluorescence (F) (*top*) and a quantitative calibration line (*bottom*) in quantitative Alu-PCR. P.C. (*left arrow*) indicates a representative positive control (a subcutaneous tumor generated from mock/HLF cells). The reaction of interest was performed using DNA from the scar-like area of cases (C; *right arrow*) to confirm that human genomic material was present in the injection site. The average amount of human genomic DNA derived from human hepatoma (mock/HLF: *n* = 3) was approximately 50 ng. Case (C) showed 55.0 pg. The genome copy number was calculated based on the fact that 1 pg corresponds to approximately 0.333 human genome copies. Correlation coefficient, *r* = −0.996

Histological changes at the injection sites and in main organs, including vessels, were examined after resection. Although the tumors that were resected at the end of the observation period no longer demonstrated suppressed growth, we examined the expression level of miR-520d-5p in the resected tumors. HMV-I tumors that received 520d-5p weekly for 12 weeks exhibited significantly upregulated 520d-5p, and HLF tumors that received 520d-5p weekly for 15 weeks tended to upregulate 520d-5p (Fig. 5a). Intratumor upregulation of 520d-5p was confirmed, but the expression levels of P53 and pluripotent markers observed in vitro were not altered relative to those in parental or scramble-transfected cells when mice were sacrificed (Additional file 6: Figure S1). HLF tumors treated with 520d/atelocollagen exhibited GFP-expressing muscle tissues, including an undifferentiated tumor that was unaccompanied by necrosis (Fig. 5b; left). In contrast, HMV-I tumors treated with 520d/atelocollagen exhibited an undifferentiated tumor accompanied by extensive necrosis (Fig. 5b; right). Approximately 0.5 mm^3^ of protruding tissue that resembled a scar at the injection site of HLF cells did not contain any malignant cells but was confirmed to be the mouse muscle subjected to the injection (Fig. 5c; top left). Interestingly, this tissue expressed GFP (Fig. 5c; bottom left). Similarly, non-tumorous tissue at the injected site of HMV-I cells did not contain any malignant cells and was histologically diagnosed as normal murine tissue (Fig. 5c; top right) but did express GFP (Fig. 5c; bottom right). In addition, adverse effects and toxicity were assessed in this study. For the period of 6 months when the mice or tumor-inoculated mice were observed, their normal activities were maintained without weight loss or anorexia. The mice did not experience any toxic reaction (such as embolism) in response to the infusion of atelocollagen (Additional file 7: Figure S2).

### Presence of human genome and gene expression at injection site of mice

By quantitative real-time Alu-PCR, we detected an average of 55.0 pg of human-derived genomic DNA in 50 ng of DNA extracted from the injection site of the mouse (Fig. 5d). This finding was usually observed in all cases in which tumors disappeared (for a case of glioblastoma, see Additional file 8: Figure S3). The site where HMV-I cells were injected and where the tumor disappeared included approximately 0.1 % human tumor cell-derived DNA in murine DNA. Alu-PCR using DNA from each organ of the mice that received intravenous injections was not successful.

**Fig. 6:**
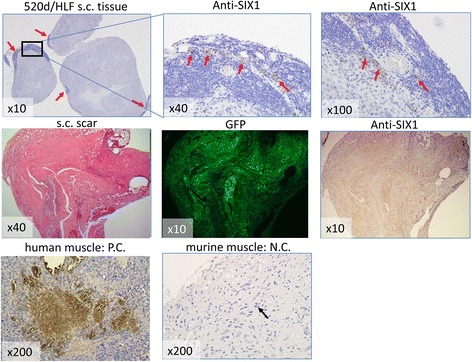
Pathological findings of human muscle-derived gene expression at the injection site. Immunohistochemistry showing human muscle-derived gene expression (SIX1) in 3 normal subcutaneous regions (*upper*) and the scar-like, slightly protruding region (*middle*). The human muscular region in subcutaneous tissue (*bottom left*) and the murine subcutaneous region (*bottom right*), including muscle, were used as a positive control and a negative control, respectively. Scar-like formation (HE stain; *middle left*) induced by injection retained GFP expression (*middle center*) and weak human SIX1 expression in murine tissue (*middle right*). The representative region for immunostaining is denoted by a rectangle (*top left*), and *arrows* denote moderate human SIX1 expression. The *arrow* shows the murine muscle region without human SIX1 staining (*bottom left*)

Immunohistochemistry revealed that murine normal subcutaneous regions (Fig. 6, upper) or the scar-like protruding region (Fig. 6, middle) at the injection site included human muscle-derived gene expression (SIX1).

## Discussion

Drug delivery carriers using RNA-based medicine have undergone repeated improvements. There is an urgent need to develop new formulations or to optimize the modification of the molecules themselves to overcome the instability of RNA molecules when they are used in biological techniques or employed for in vivo studies to evaluate their usefulness [32–34].

Increasing evidence has suggested that miRNAs are strongly associated with numerous biological processes, including oncogenesis or tumor progression [35–37]. Improvements in nanoparticles [38], cationic polymer [39], antisense [32] or mimic RNA [40, 41], viruses [13], and artificial viruses [42] have been intensely pursued to increase quality. Atelocollagen is also a DDS carrier that is used topically in cosmetics and in plastic and reconstructive surgery. This biomaterial is now experiencing expanded use for intraperitoneal or systemic administration and for subcutaneous injection in cancer therapy [23, 43–46].

In this study, we first attempted to examine the tumor suppressive effect of miR-520d-5p using atelocollagen with subcutaneous injection. Because this DDS carrier could not penetrate into all cancer cells and left viable cancer cells in tumor tissues, it allowed the tumor to grow gradually, indicating that an assay system must be established to optimize the injection conditions. It was reported previously that atelocollagen accumulates in prostate cancer cells (PC-3) [47]. However, when 520d/atelocollagen was injected into subcutaneous tumors, the tumor size did not exceed 8 mm for the cells used in this study (HLF and HMV-I), and tumor growth was significantly suppressed each week. Upregulation of p53 and Nanog and downregulation of AICDA were observed, similar to observations for other undifferentiated cancer cells, e.g., glioblastoma, pancreatic cancer and anaplastic thyroid cancer. The in vitro study could not significantly confirm intratumoral expression after sacrifice compared with parental or scrambled-transfected cells. Although a considerable number of mice did not develop any tumor nodules, the greatest effect of 520d/atelocollagen was the suppression of metastasis. When 520d/atelocollagen was injected around the tumor rather than intratumorally, mice in which a tumor-inhibiting effect was observed did not display any (micro) metastases, even in the tissues surrounding the tumor. The inoculation of 520d-5p-expressing HMV-I (520d/HMV-I) resulted in the suppression of tumor formation in 33.3 % of mice (3/9), and metastasis was inhibited in 100 % (9/9) of mice (Additional file 9: Table S4). We cannot exclude the possibility that mouse cells and human cells may co-exist in the mouse tissue without evidence of rejection. A similar phenomenon may exist in 520d-expressing human glioblastoma (T98G) cells that were intracranially injected into the thalamus, resulting in the co-existence of human-derived cancer cells as endothelial cells and glial cells (i.e., normal human cells) in the brain tissue of mice who received injection without generating a malignant brain tumor (Additional file 8: Figure S3). Furthermore, we examined the transcriptional expression levels of BRAF, PDCD1 and PDCD1 LG2 to investigate their correlation with gene expression. As a selective BRAF inhibitor, vemurafenib is used for patients with malignant melanoma [47]. BRAF tended to be downregulated by 520d-5p (*P* = 0.05 by the Mann-Whitney *U* test) (Additional file 10: Figure S4). In addition, 520d-5p may have other anti-cancer effects on BRAF-related genes, except for the conversion of transfectants to a more immature status.

Atelocollagen prevents immune-stimulatory adverse effects [48], and 520d-5p suppresses immunoreaction by downregulating AICDA [27]. These properties may be involved in the phenomenon observed in this study. Thus, miR-520d may be an interesting molecule whose mechanism should be clarified.

We postulate that a possible mechanism underlying tumor-suppressing effect by 520d-5p can be TP53 upregulation induced by downregulation of MYST1 which is implicated in methylation process [49]. Because MYST1 are indirectly downregulated by 520d-5p upregulation, we are attempting to prove the detail mechanisms.

Thus, 520d/atelocollagen may be a potent candidate therapeutic agent with anti-metastatic effects [50]. Because the efficiency of the introduction of such conjugating materials into a target cell or target tissue remains unclear, further careful investigation is needed before the application of this therapy in humans.

## Conclusions

To our knowledge, this study provides the first evidence that the 520d-5p/atelocollagen vehicle is a useful biomaterial for in vivo xenograft studies or for clinical use as an anti-cancer therapy.

## Abbreviations

AICDA, activation-induced cytidine deaminase; DDS(s), drug delivery system(s); ds, double-stranded; FBS, fetal bovine serum; GFP, green fluorescent protein; HE, hematoxylin-eosin; hsa-miR, homosapiens microRNA; hTERT, human telomerase reverse transcriptase; ncRNA, noncoding RNA; PBS, phosphate-buffered saline; RPMI, Roswell Park Memorial Institute; RT-PCR, reverse transcription-polymerase chain reaction; s.c., subcutaneous(ly); sh, short hairpin; si, small interfering; UTRs, untranslated regions

## Additional files

Additional file 1: Table S1. The sequences of primers used in this study. Primers used in this study are presented. (PDF 69 kb) Additional file 2: Figure S5. A representative case 24 weeks after subcutaneous injection. A pathologist recognized adipose tissue and skeletal muscle thought to be the existing structure but could not confirm the clear tumor composition at the injection site (HE stain; top left, the perspective of the specimen; bottom, x40, x100 magnification). We did not identify any macroscopic tumors in this case. (PDF 341 kb) Additional file 3: Table S2. The suppression rate (%) of tumor volume. The suppression rate (%) of tumor volume compared with the control each week is shown in Table 1. Greater than 85 % suppression 6 weeks after administration was observed in a xenograft model by subcutaneous injection. (PDF 92 kb) Additional file 4: Table S3. Effective rate of tumor disappearance. Effective rate (%) of tumor disappearance by systemic administration compared with that of the control is shown in Table 1. Greater than 75 % of the tumor cells disappeared based on macroscopic and microscopic observation. (PDF 94 kb) Additional file 5: Figure S6. Representative gene expression in HMV-I tumors without suppression. When the animals were sacrificed, most tumors grew increasingly and the suppressive effect of 520d-5p on tumor growth could not be observed by RT-PCR. *: *P* < 0.05 by Man-Whitney *U* test. (PDF 267 kb) Additional file 6: Figure S1. A representative gene expression profile of 520d/HMV-I. Upregulation of p53 and Nanog and downregulation of AICDA were observed in 520d/HMV-I cells in vitro, similar to the results for 520d/HLF cells. *, *P* < 0.05 by one-way ANOVA. Oct4 was not significantly upregulated, but it was weakly upregulated, as indicated by western blotting. (PDF 81 kb) Additional file 7: Figure S2. The side effects of 520d/atelocollagen and the presence of tumor cells in the inoculated tumor in seven organs. Neither the presence of embolism induced by atelocollagen or evidence of micrometastases was detected pathologically in brain, liver, spleen, heart, lung, small intestine and kidney. (PDF 185 kb) Additional file 8: Figure S3. An in vivo study using glioblastoma cells (T98G) treated with miR-520d-5p (520d/T98G). Three surviving mice were examined for human-derived gene expression in murine brain tissue 3 months after intracranial injection. **a** KSN/Slc mice were anesthetized with sodium pentobarbital (50 mg/kg intraperitoneally) and placed in a stereotaxic apparatus. During surgery, the animals’ body temperature was maintained at 37 °C using a heating pad. The skull was exposed, and a small craniotomy was made over the left striatum. A 30-gauge injection needle connected to a 10-μl Hamilton syringe through polyethylene tubing was used for 520d/T98G cell transplantation. The injection needle was inserted stereotaxically into the left striatum (A 2.0 mm, L 0.5 mm, D 1.2 mm from bregma) (left), and 1 μl of cell suspension (1 × 10^8^ cells/μl) was pressure-injected (right). After injection, the needle was slowly withdrawn, and the skull hole was covered with dental cement. The incision was sutured with 6-0 Prolene. After recovery from surgery, the animals were returned to their home cage. **b** Immunohistochemistry was performed using anti-hGFAP antibody, and human-derived GFAP protein expression was confirmed in glial cells and vascular endothelial cells in the murine thalamus (left to right: x40, x200, x400 magnification). (PDF 203 kb) Additional file 9: Table S4. Subcutaneous changes of 520d-5p-expressing HMV-I in a xenograft model. Subcutaneous changes of 520d-5p-expressing HMV-I in a xenograft model were recorded as criteria for tumor formation and metastasis. In HMV-I, the disappearance rate of tumors was greater than the outcome (12.5 %) of therapeutic administration in vivo. (PDF 32 kb) Additional file 10: Figure S4. Relative mRNA expression levels of BRAF, PDCD1 and PDCD1LG2, standardized to β-actin in HMV-I cells. A box plot was drawn to compare expression levels. BRAF was not significantly downregulated in 520d/HMV-I cells compared with mock/HMV-I cells (by Mann-Whitney *U* test, *n* = 3), but it tended to be downregulated by 520d-5p. *, *P* < 0.05. (PDF 89 kb)
