# Correction to: Tumor-suppressive effects of atelocollagen-conjugated hsa-miR-520d-5p on un-differentiated cancer cells in a mouse xenograft model

**DOI:** 10.1186/s12885-017-3653-2

**Published:** 2017-10-02

**Authors:** Yoshitaka Ishihara, Satoshi Tsuno, Satoshi Kuwamoto, Taro Yamashita, Yusuke Endo, Keigo Miura, Yugo Miura, Takemasa Sato, Junichi Hasegawa, Norimasa Miura

**Affiliations:** 1 Division of Pharmacotherapeutics, Department of Pathophysiological & Therapeutic Science, Faculty of Medicine, Tottori University, 86 Nishicho, Yonago, Tottori 683-8503 Japan; 2 Division of Molecular Pathology, Faculty of Medicine, Tottori University, 86 Nishicho, Yonago, Tottori 683-8503 Japan; 3 Department of Gastroenterology, Tottori University Hospital, 86 Nishicho, Yonago, Tottori 683-8504 Japan; 4 PEZY-Pharma, 86 Nishicho, Yonago, Tottori 683-8503 Japan; 5 Orthopedic Surgery, Tokyo Medical and Dental University, 1-5-45 Yushima, Bunkyo-ku, Tokyo 113-8510 Japan; 6 Division of Neurobiology, School of Life Science, Faculty of Medicine, Tottori University, 86 Nishicho, Yonago, Tottori 683-8503 Japan

## Correction

After publication of the original article [1] the authors found the following errors had occurred:

1. The imaging photo was incorrectly selected for Fig. 4. The original article contains imaging relating to another series. An updated version of Fig. 4 is included with this Correction.
2. In the original Fig. 5b figure legend, reference is made to an HE stain, however this has been made incorrectly. All references to an HE stain should therefore be omitted. The entirety of Figure legend 5b is included below, with the amendments made to the first sentence (which is included in bold for reference):

**Fig. 4:**
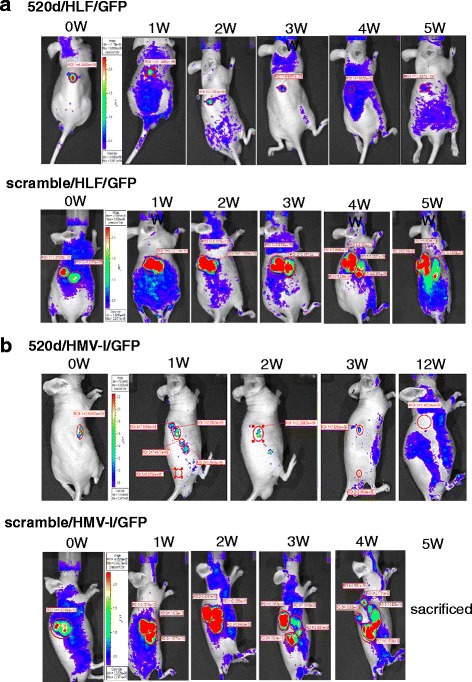
The therapeutic effect of 520d-5p-conjugated atelocollagen on subcutaneously inoculated tumors as monitored by an in vivo imaging system. **a** A representative image of the therapeutic effect of 520d-5p/atelocollagen using an in vivo imaging system. At each inoculation site, 37.5% (3/8) of the tumors disappeared. The growth of tumors that received 520d-5p was significantly suppressed, and metastatic ability was entirely inhibited in the remaining mice. A time course (*top*) of 520d/HLF/GFP cells (520d-5p-transfected and GFP-expressing HLF) is depicted. Representative sequential images (*bottom*) of scrambled/HLF/GFP cells (scrambled-transfected GFP-expressing HLF). **b** In HMV-I tumors, 12.5% (1/8) of the tumors disappeared. After treatment with 520d-5p, the growth of other tumors was significantly suppressed, and their metastatic ability was entirely inhibited. 520d/HMV-I/GFP and scrambled/HMV-I/GFP indicate 520d-5p-transfected, GFP-expressing HLF (*top*) and scramble-transfected, GFP-expressing HMV-I (*bottom*), respectively

**Fig. 5:**
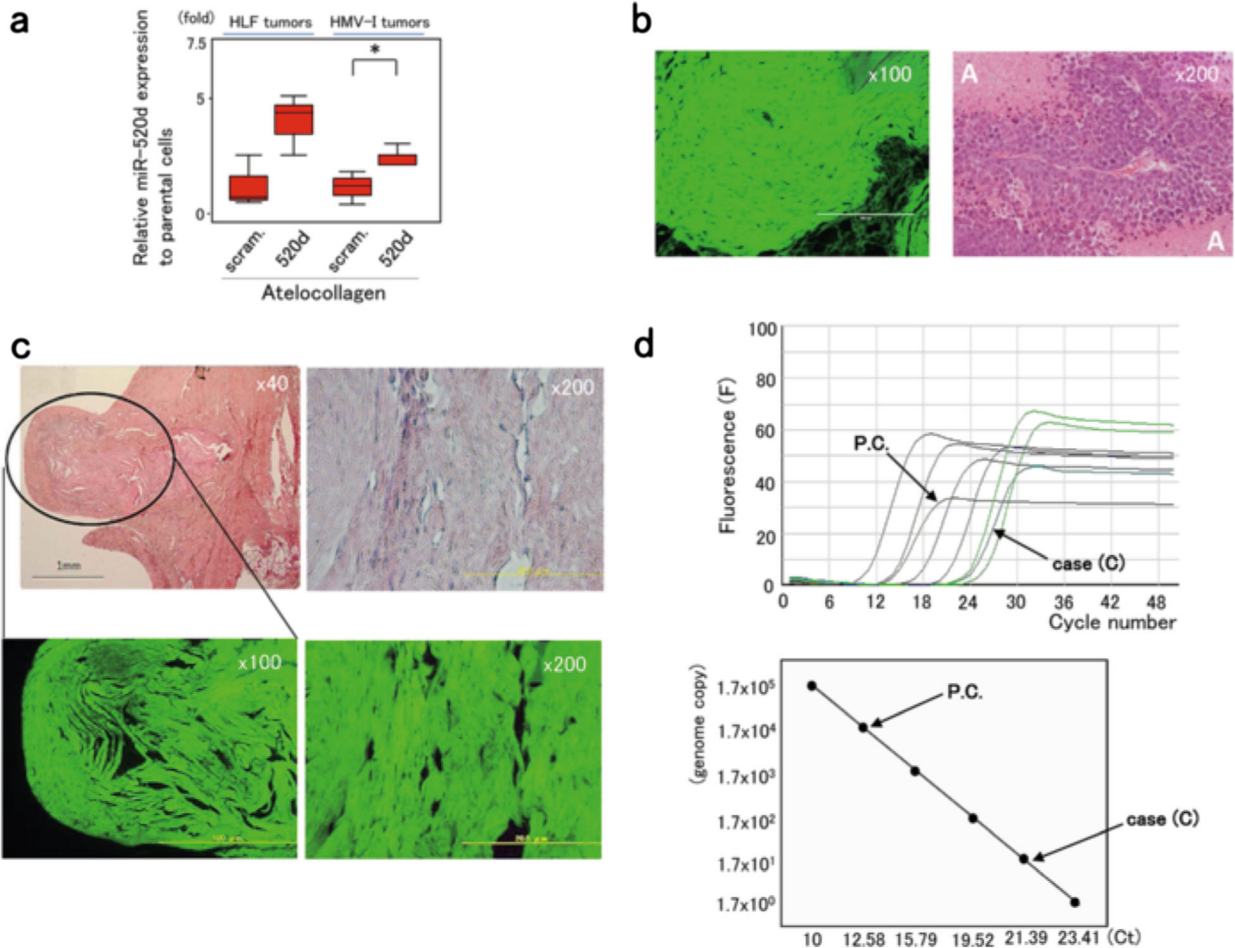
Evidence of human genomic DNA at the injection site. **a** miR-520d-5p expression was examined in HLF (*n* = 5) and HMV-I (*n* = 7) tumors resected after 520d/atelocollagen treatment. 520d/HMV-I tumors significantly expressed 520d-5p (*, *P* < 0.05), and 520d/HLF tumors tended to express miR-520d-5p (*P* = 0.051). scram.: scrambled. **b** Microscopic examination of HLF (left; ×100 magnification) and HMV-I tumors (right; ×200 magnification) that displayed suppressed tumor growth. HLF tumors treated with 520d/atelocollagen exhibited GFP-expressing muscle tissues, including undifferentiated tumor cells unaccompanied by necrosis. HMV-I tumors treated with 520d/atelocollagen showed undifferentiated neoplastic cells. This finding is not inconsistent, as malignant melanomas are observed to be accompanied by extensive necrosis, as shown around A. **c** Histological findings in the area where there should have been a tumor are presented (HE stain, ×40 magnification). The area of the HLF tumors that disappeared is surrounded by an oval (*left top*). GFP expression was confirmed (*left bottom*). Histological findings in the disappearance site of HMV-I tumors showed no malignant cells. This finding was similar to that for 520d–treated HLF tumors (HE stain, ×200 magnification) (*right top*), but we observed GFP even in the connective tissue (*right bottom*). We did not observe any malignant cells in the injection sites of mice in either case. **d** Fluorescence (F) (*top*) and a quantitative calibration line (*bottom*) in quantitative Alu-PCR. P.C. (*left arrow*) indicates a representative positive control (a subcutaneous tumor generated from mock/HLF cells). The reaction of interest was performed using DNA from the scar-like area of cases (C; *right arrow*) to confirm that human genomic material was present in the injection site. The average amount of human genomic DNA derived from human hepatoma (mock/HLF: *n* = 3) was approximately 50 ng. Case (C) showed 55.0 pg. The genome copy number was calculated based on the fact that 1 pg corresponds to approximately 0.333 human genome copies. Correlation coefficient, *r* = −0.996

**Figure legend 5b:**

**Microscopic examination of HLF (left; ×100 magnification) and HMV-I tumors (** ***right*** **; ×200 magnification) that displayed suppressed tumor growth.** HLF tumors treated with 520d/atelocollagen exhibited GFP-expressing muscle tissues, including undifferentiated tumor cells unaccompanied by necrosis. HMV-I tumors treated with 520d/atelocollagen showed undifferentiated neoplastic cells. This finding is not inconsistent, as malignant melanomas are observed to be accompanied by extensive necrosis, as shown around A.

A corrected version of Fig. 5 is included with this Correction.
