# Supplementary material for: Tumor-suppressive effects of atelocollagen-conjugated hsa-miR-520d-5p on un-differentiated cancer cells in a mouse xenograft model
Source: BMC Cancer. 2016 Jul 7;16:415. doi: 10.1186/s12885-016-2467-y (PMC4936056; doi:10.1186/s12885-016-2467-y)
Supplement: Additional file 1: Table S1. — The sequences of primers used in this study. Primers used in this study are presented. (PDF 69 kb) [file 12885_2016_2467_MOESM1_ESM.pdf]

Table S1

Primers used in this study

Gene	Sense	Antisense	product size (bp)
β-actin	ACCTGACTGACTACCTCATG	GCAGCCGTGGCCATCTCTTG	146
Oct4	CGGAAAGAGAAAGCGAACCA	CGGACCACATCCTTCTCCAG	135
Nanog	CAGAAGGCCTCAGCACCTAC	ACTGGATGTTCTGGGTCTGG	145
P53	GCTTCGAGATGTTCCGAGAG	TTATGGCGGGAGGTAGACTG	133
AICDA	CGTAGTGAAGAGGCGTGACA	TGTAGCGGAGGAAGAGCAAT	102
miR-520d-5p	TCTACAAAGGGAAGCCCTTTCTG		
