# Supplementary material for: Tumor-suppressive effects of atelocollagen-conjugated hsa-miR-520d-5p on un-differentiated cancer cells in a mouse xenograft model
Source: BMC Cancer. 2016 Jul 7;16:415. doi: 10.1186/s12885-016-2467-y (PMC4936056; doi:10.1186/s12885-016-2467-y)

Fig. S5

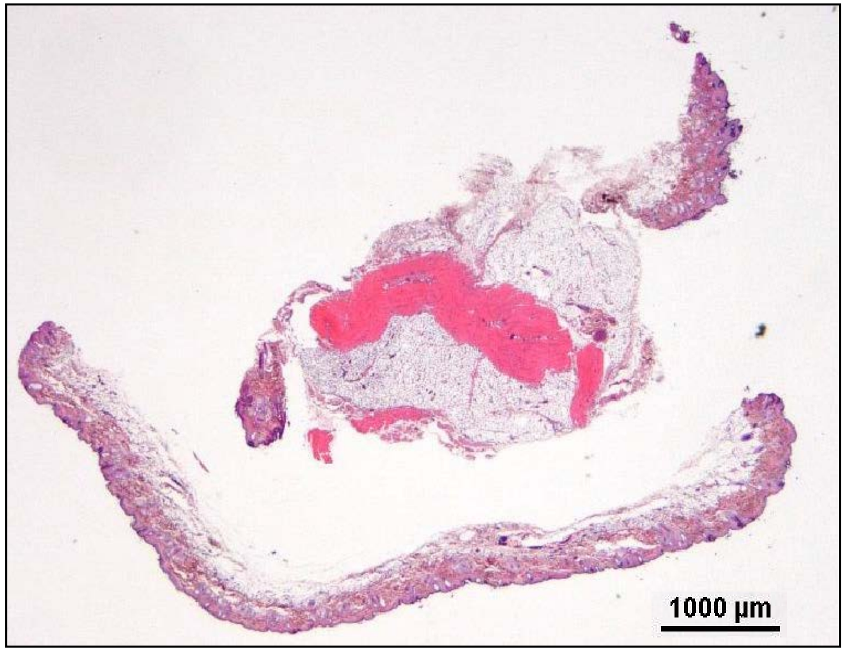

A representative case 24 weeks later subcutaneous injection.
Pathologists recognized adipose tissue and a skeletal muscle thought to be the existing structure and cannot confirm the clear tumor ingredient at the injection site. We could not find any tumor macroscopically in this case.

X40 s.c.

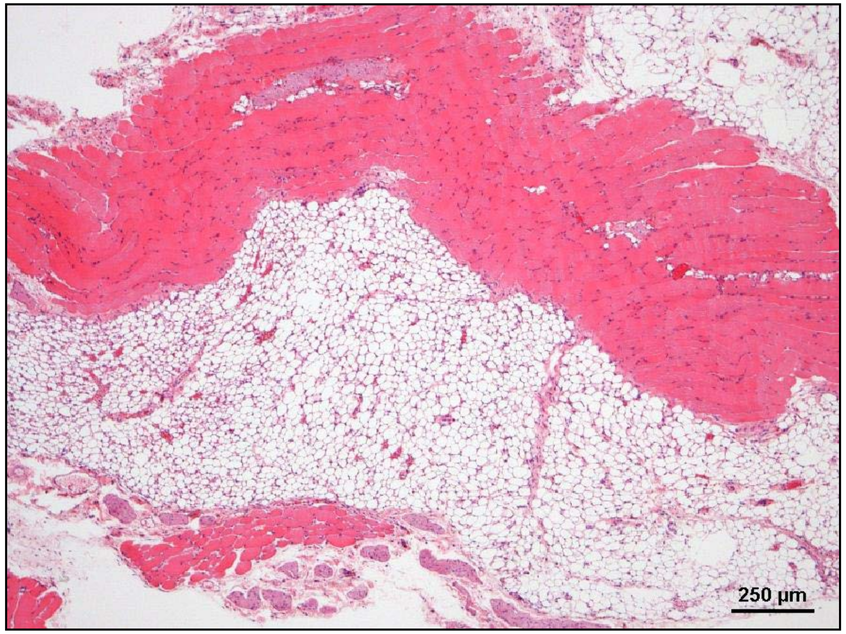

X100 s.c.

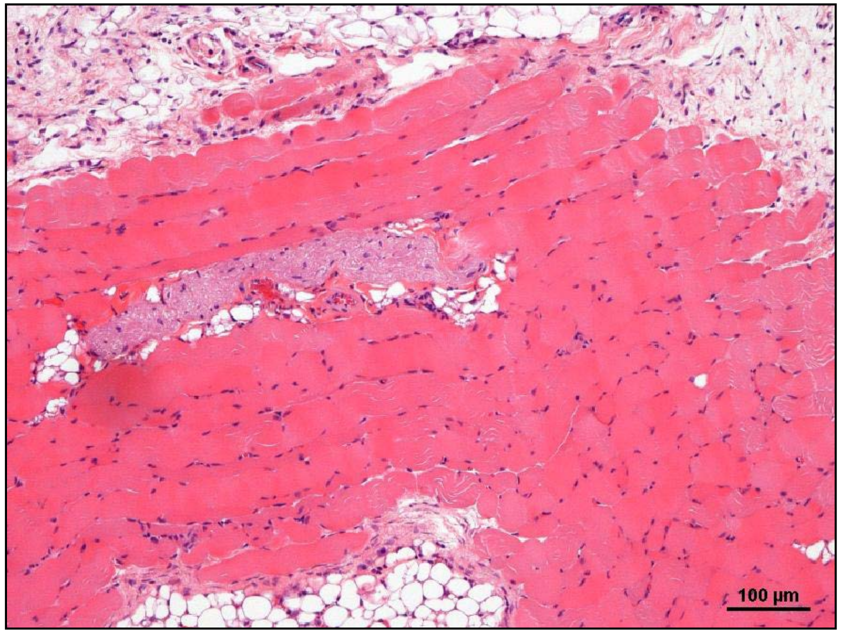

Supplement: Additional file 2: Figure S5. — A representative case 24 weeks after subcutaneous injection. A pathologist recognized adipose tissue and skeletal muscle thought to be the existing structure but could not confirm the clear tumor composition at the injection site (HE stain; top left, the perspective of the specimen; bottom, x40, x100 magnification). We did not identify any macroscopic tumors in this case. (PDF 341 kb) [file 12885_2016_2467_MOESM2_ESM.pdf]
