# Supplementary material for: Tumor-suppressive effects of atelocollagen-conjugated hsa-miR-520d-5p on un-differentiated cancer cells in a mouse xenograft model
Source: BMC Cancer. 2016 Jul 7;16:415. doi: 10.1186/s12885-016-2467-y (PMC4936056; doi:10.1186/s12885-016-2467-y)
Supplement: Additional file 3: Table S2. — The suppression rate (%) of tumor volume. The suppression rate (%) of tumor volume compared with the control each week is shown in Table 1. Greater than 85 % suppression 6 weeks after administration was observed in a xenograft model by subcutaneous injection. (PDF 92 kb) [file 12885_2016_2467_MOESM3_ESM.pdf]

Table S2

Suppression rate (%) of tumor volume compared with control in each week

cancer cells	5W	6W	7W	8W	9W	10W
HLF	66.6	86.9	86.9	90.6	94.0	92.7
HMV-I	84.1	85.5	90	89.1	91.7	85.9
