# Supplementary material for: Tumor-suppressive effects of atelocollagen-conjugated hsa-miR-520d-5p on un-differentiated cancer cells in a mouse xenograft model
Source: BMC Cancer. 2016 Jul 7;16:415. doi: 10.1186/s12885-016-2467-y (PMC4936056; doi:10.1186/s12885-016-2467-y)
Supplement: Additional file 4: Table S3. — Effective rate of tumor disappearance. Effective rate (%) of tumor disappearance by systemic administration compared with that of the control is shown in Table 1. Greater than 75 % of the tumor cells disappeared based on macroscopic and microscopic observation. (PDF 94 kb) [file 12885_2016_2467_MOESM4_ESM.pdf]

Table S3

Effective rate (%) of tumor disappearance by systemic administration, compared with that of control

cancer cells	Presence of intraperitoneal tumorous nodules		Effective rate −/total number (%)
	+	−	
HLF	2	6	75.0
HMV-I	1	7	87.5
