# Supplementary material for: Tumor-suppressive effects of atelocollagen-conjugated hsa-miR-520d-5p on un-differentiated cancer cells in a mouse xenograft model
Source: BMC Cancer. 2016 Jul 7;16:415. doi: 10.1186/s12885-016-2467-y (PMC4936056; doi:10.1186/s12885-016-2467-y)
Supplement: Additional file 5: Figure S6. — Representative gene expression in HMV-I tumors without suppression. When the animals were sacrificed, most tumors grew increasingly and the suppressive effect of 520d-5p on tumor growth could not be observed by RT-PCR. *: P < 0.05 by Man-Whitney U test. (PDF 267 kb) [file 12885_2016_2467_MOESM5_ESM.pdf]

Fig. S6

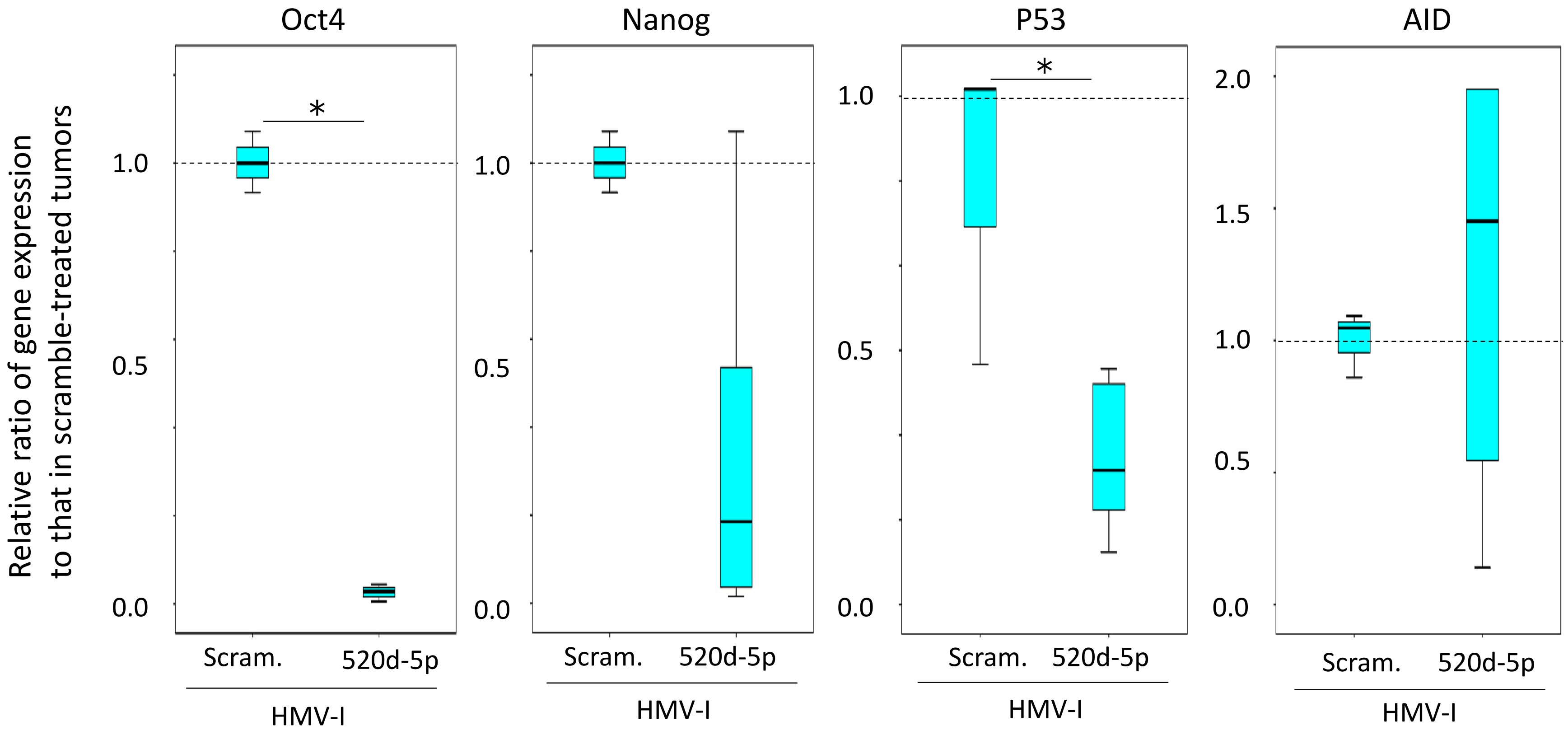

When the animals were sacrificed, most tumors grew increasingly and the suppressive effect of 520d-5p on tumor growth could not be observed by RT-PCR.

＊: $P<0.05$ by Man-Whitney *U* test
