# Supplementary material for: Tumor-suppressive effects of atelocollagen-conjugated hsa-miR-520d-5p on un-differentiated cancer cells in a mouse xenograft model
Source: BMC Cancer. 2016 Jul 7;16:415. doi: 10.1186/s12885-016-2467-y (PMC4936056; doi:10.1186/s12885-016-2467-y)
Supplement: Additional file 6: Figure S1. — A representative gene expression profile of 520d/HMV-I. Upregulation of p53 and Nanog and downregulation of AICDA were observed in 520d/HMV-I cells in vitro, similar to the results for 520d/HLF cells. *, P < 0.05 by one-way ANOVA. Oct4 was not significantly upregulated, but it was weakly upregulated, as indicated by western blotting. (PDF 81 kb) [file 12885_2016_2467_MOESM6_ESM.pdf]

Fig. S1

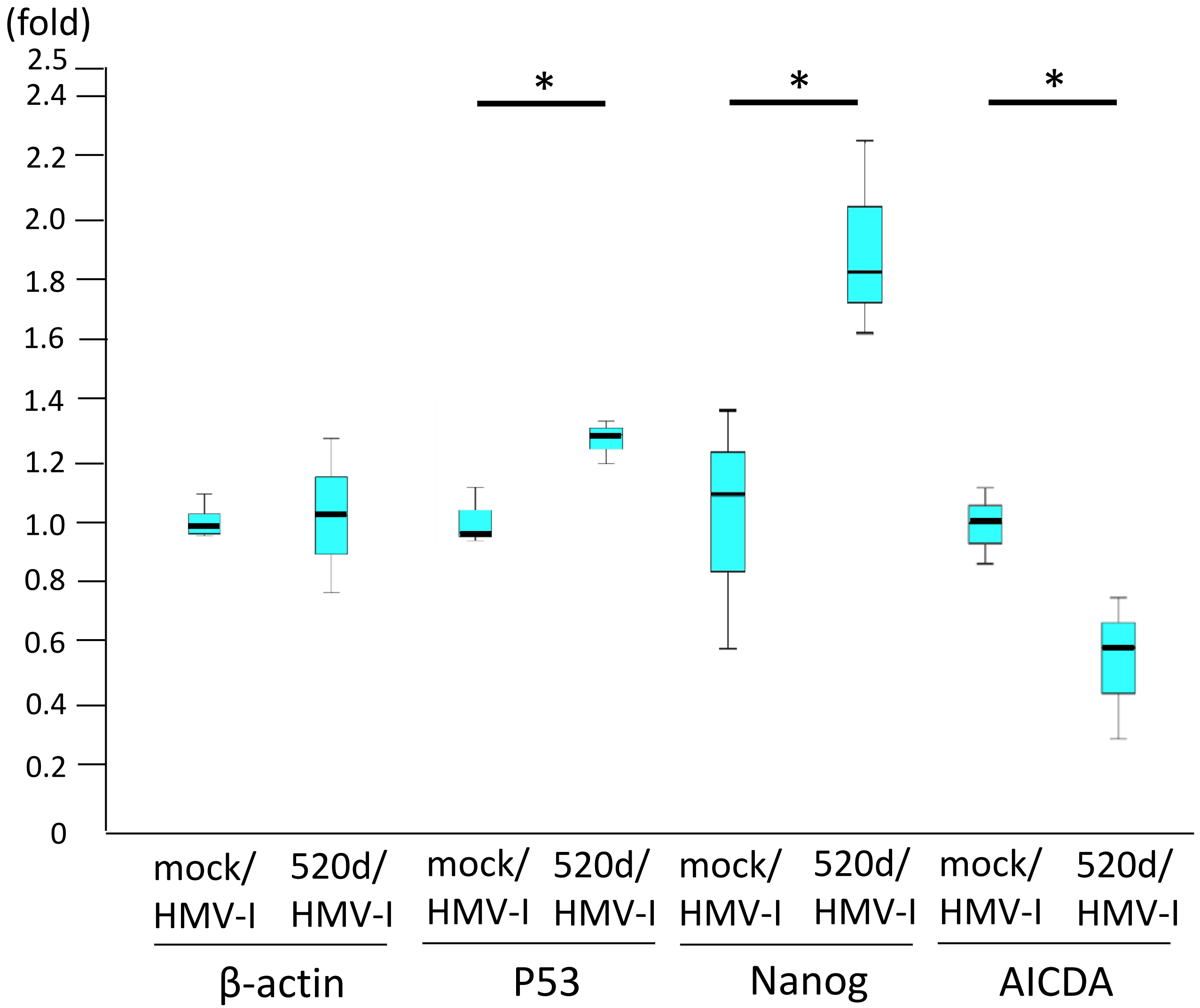

Representative gene expression profile in 520d/HMV-I is shown. Upregulation of P53 and Nanog, and downregulation of AICDA were observed in 520d/HMV-I *in vitro*, similar to those in 520d/HLF. *: P<0.05 by one way ANOVA. Oct4 was not significantly upregulated, but weakly upregulated in western blotting.
