# Supplementary material for: Tumor-suppressive effects of atelocollagen-conjugated hsa-miR-520d-5p on un-differentiated cancer cells in a mouse xenograft model
Source: BMC Cancer. 2016 Jul 7;16:415. doi: 10.1186/s12885-016-2467-y (PMC4936056; doi:10.1186/s12885-016-2467-y)
Supplement: Additional file 7: Figure S2. — The side effects of 520d/atelocollagen and the presence of tumor cells in the inoculated tumor in seven organs. Neither the presence of embolism induced by atelocollagen or evidence of micrometastases was detected pathologically in brain, liver, spleen, heart, lung, small intestine and kidney. (PDF 185 kb) [file 12885_2016_2467_MOESM7_ESM.pdf]

**Fig. S2**

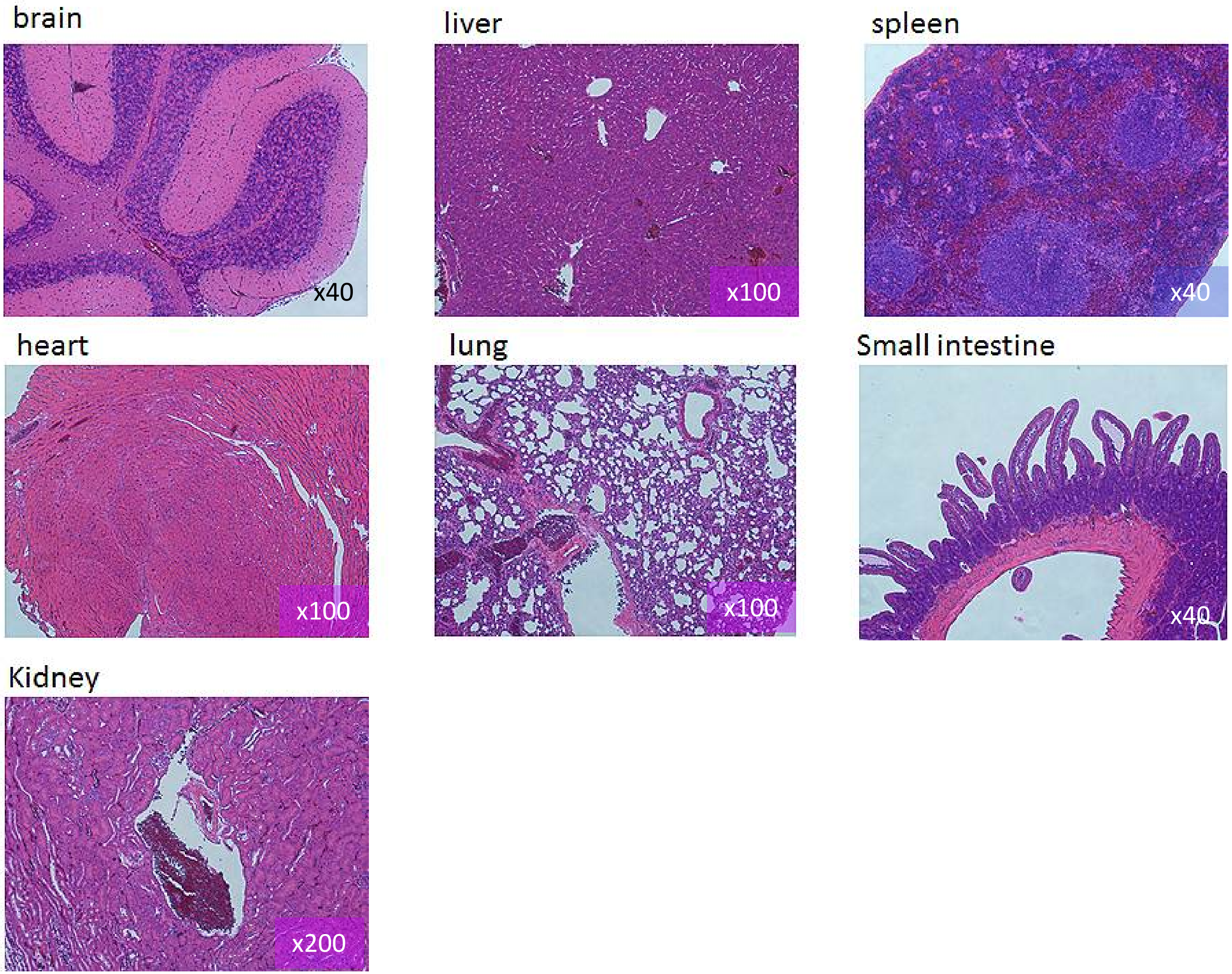


The side effect of 520d/Atelocollagen and the presence of tumor cells by inoculated tumor were examined in seven organs (brain, liver, spleen, heart, lung, small intestine and kidney). The presence of embolism by Atelocollagen or the evidences of micrometastasis could not be found at all pathologically.
