# Supplementary material for: Tumor-suppressive effects of atelocollagen-conjugated hsa-miR-520d-5p on un-differentiated cancer cells in a mouse xenograft model
Source: BMC Cancer. 2016 Jul 7;16:415. doi: 10.1186/s12885-016-2467-y (PMC4936056; doi:10.1186/s12885-016-2467-y)
Supplement: Additional file 8: Figure S3. — An in vivo study using glioblastoma cells (T98G) treated with miR-520d-5p (520d/T98G). Three surviving mice were examined for human-derived gene expression in murine brain tissue 3 months after intracranial injection. a KSN/Slc mice were anesthetized with sodium pentobarbital (50 mg/kg intraperitoneally) and placed in a stereotaxic apparatus. During surgery, the animals’ body temperature was maintained at 37 °C using a heating pad. The skull was exposed, and a small craniotomy was made over the left striatum. A 30-gauge injection needle connected to a 10-μl Hamilton syringe through polyethylene tubing was used for 520d/T98G cell transplantation. The injection needle was inserted stereotaxically into the left striatum (A 2.0 mm, L 0.5 mm, D 1.2 mm from bregma) (left), and 1 μl of cell suspension (1 × 10^8^ cells/μl) was pressure-injected (right). After injection, the needle was slowly withdrawn, and the skull hole was covered with dental cement. The incision was sutured with 6-0 Prolene. After recovery from surgery, the animals were returned to their home cage. b Immunohistochemistry was performed using anti-hGFAP antibody, and human-derived GFAP protein expression was confirmed in glial cells and vascular endothelial cells in the murine thalamus (left to right: x40, x200, x400 magnification). (PDF 203 kb) [file 12885_2016_2467_MOESM8_ESM.pdf]

Fig. S3

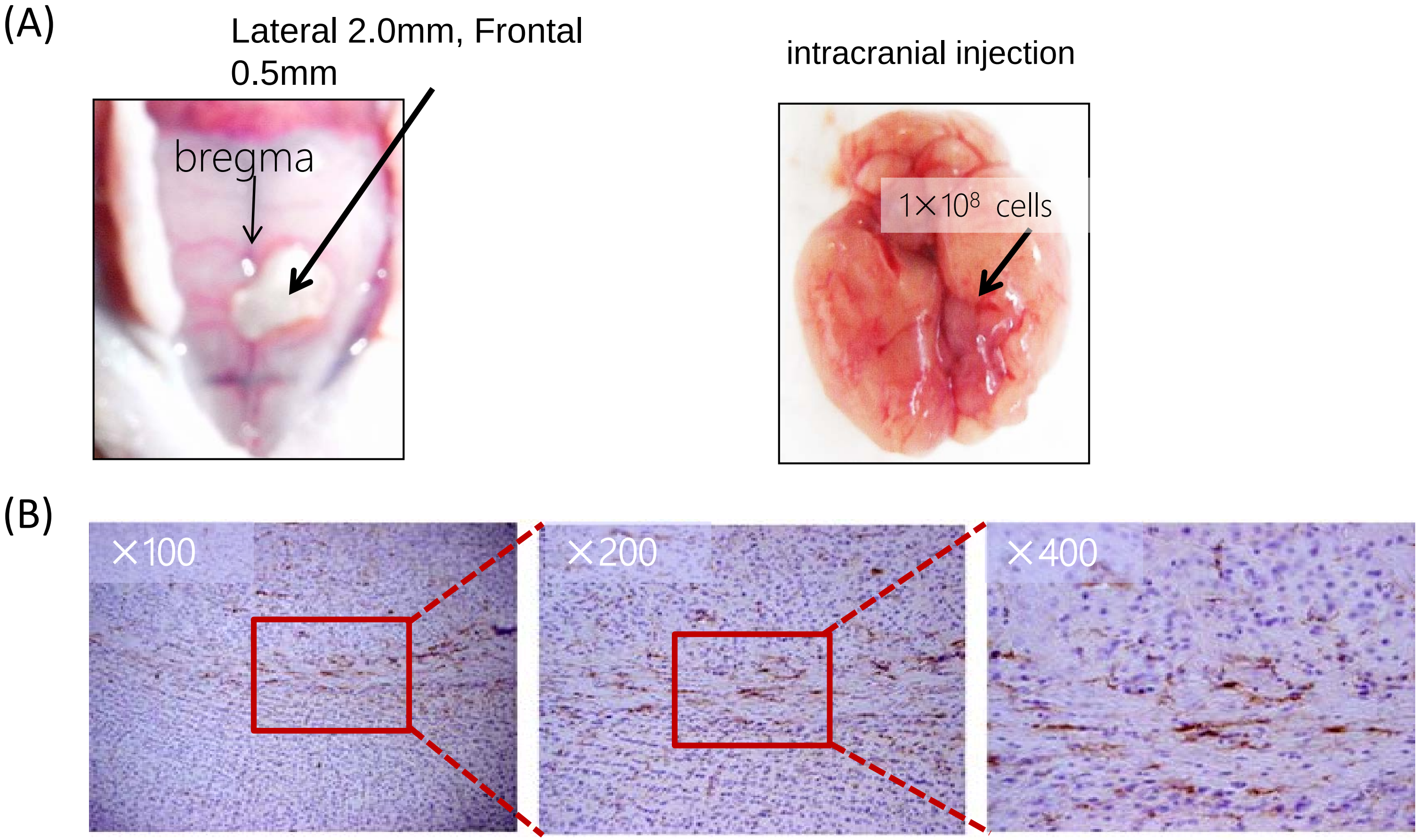

In vivo study using glioblastoma cells (T98G) treated with miR-520d-5p (520d/T98G) was performed (n=6). Three survived mice were examined the human-derived gene expression in murine brain tissue after 3 month later than intracranial injection. (A) KSN/Slc were anesthetized with sodium pentobarbital (50 mg/kg, i.p.) and placed in a stereotaxic apparatus. During surgery, animal's body temperature was kept at 37° C using a heating pad. The skull was exposed and a small craniotomy was made over the left striatum. A 30G injection needle connected to a 10 μl Hamilton syringe through polyethylene tubing was used for 520d/T98G cell transplantation. Injection needle was inserted stereotaxically into the left striatum (A 2.0 mm, L 0.5 mm, D 1.2 mm from bregma) (left) and 1 μl of cell suspension ($1x10^8$ cells/μl) was pressure-injected (right). After injection, the needle was slowly withdrawn and the skull hole was covered with dental cement. The incision was sutured with 6-0 Prolene. After recovery from surgery, animals were returned to their home cage. (B) Immunohistochemistry was performed using anti-hGFAP antibody, resulting that human-derived GFAP protein expression was confirmed as a part of glial cells and vascular endothelial cells in murine thalamus (left to right; x40, x200, x400).
