# Supplementary material for: Tumor-suppressive effects of atelocollagen-conjugated hsa-miR-520d-5p on un-differentiated cancer cells in a mouse xenograft model
Source: BMC Cancer. 2016 Jul 7;16:415. doi: 10.1186/s12885-016-2467-y (PMC4936056; doi:10.1186/s12885-016-2467-y)
Supplement: Additional file 9: Table S4. — Subcutaneous changes of 520d-5p-expressing HMV-I in a xenograft model. Subcutaneous changes of 520d-5p-expressing HMV-I in a xenograft model were recorded as criteria for tumor formation and metastasis. In HMV-I, the disappearance rate of tumors was greater than the outcome (12.5 %) of therapeutic administration in vivo. (PDF 32 kb) [file 12885_2016_2467_MOESM9_ESM.pdf]

Table S4

Subcutaneous changes of 520d-5p-expressing HMV-I in a xenografted model

	+	−
tumor formation	6/9 (66.7%)	3/9 (33.3%)
metastasis	0/9 (0%)	9/9 (100%)

6/9 (66.7%) was suppressed the growth, compared with mock-transfected cells or parental cells
