# Supplementary material for: Tumor-suppressive effects of atelocollagen-conjugated hsa-miR-520d-5p on un-differentiated cancer cells in a mouse xenograft model
Source: BMC Cancer. 2016 Jul 7;16:415. doi: 10.1186/s12885-016-2467-y (PMC4936056; doi:10.1186/s12885-016-2467-y)
Supplement: Additional file 10: Figure S4. — Relative mRNA expression levels of BRAF, PDCD1 and PDCD1LG2, standardized to β-actin in HMV-I cells. A box plot was drawn to compare expression levels. BRAF was not significantly downregulated in 520d/HMV-I cells compared with mock/HMV-I cells (by Mann-Whitney U test, n = 3), but it tended to be downregulated by 520d-5p. *, P < 0.05. (PDF 89 kb) [file 12885_2016_2467_MOESM10_ESM.pdf]

Fig. S4

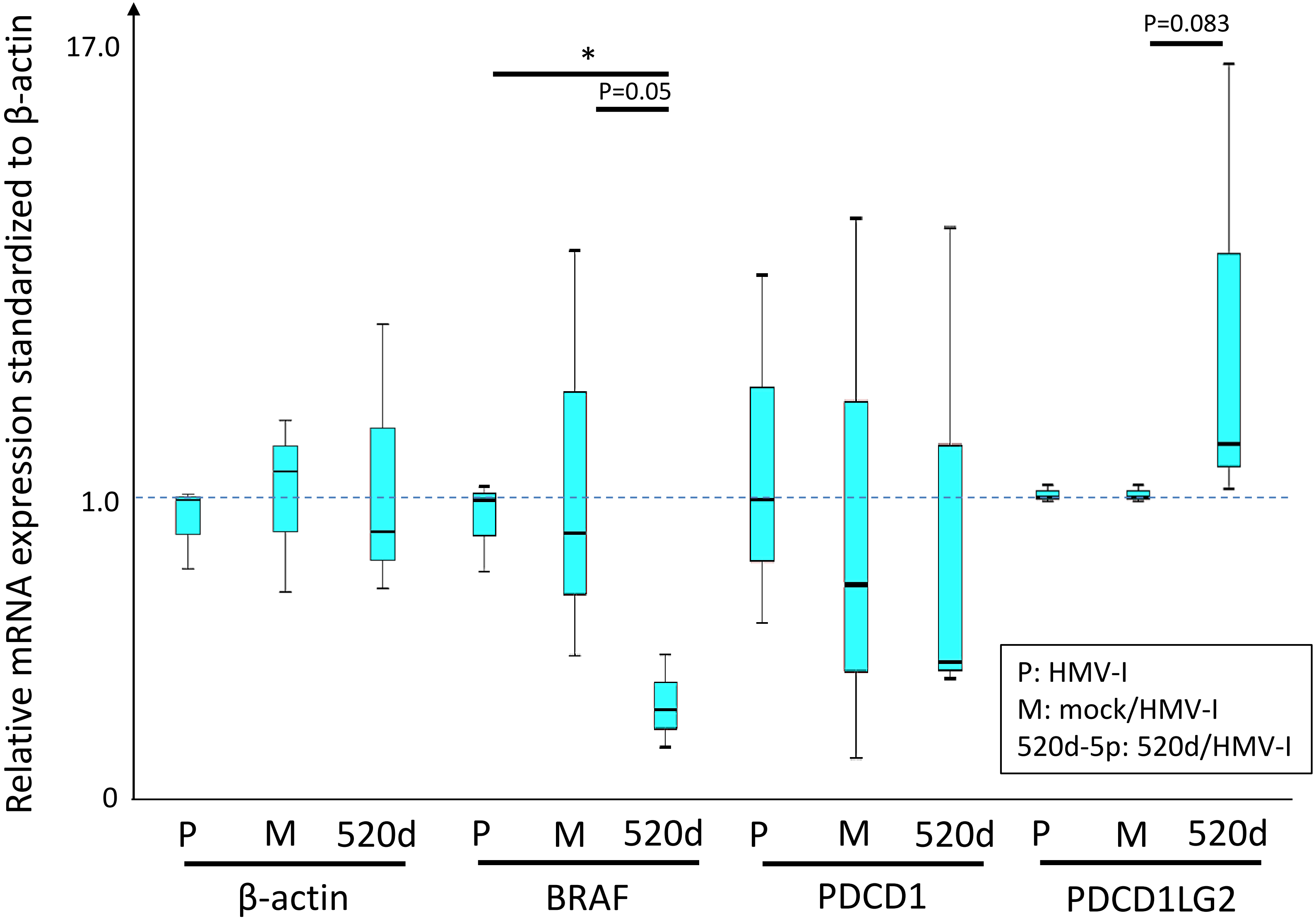

Relative mRNA expression of BRAF, PDCD1 and PDCD1LG2 standardized to β-actin were shown. Box plot was drawn compared with the expression in HMV-I cells. BRAF was not significantly downregulated in 520d/HMV-I, compared with mock/HMV-I (by Mann-Whitney *U* test, n=3), but it tended to be downregulated by 520d-5p. *: P<0.05.
